# PAR2 Participates in the Development of Cough Hypersensitivity in Guinea Pigs by Regulating TRPA1 Through PKC

**DOI:** 10.3390/biom15020208

**Published:** 2025-02-01

**Authors:** Yiqing Zhu, Tongyangzi Zhang, Haodong Bai, Wanzhen Li, Shengyuan Wang, Xianghuai Xu, Li Yu

**Affiliations:** Department of Pulmonary and Critical Care Medicine, Tongji Hospital, School of Medicine, Tongji University, Shanghai 200065, China

**Keywords:** chronic cough, cough hypersensitivity, PAR2, TRPA1, whole-cell patch-clamp

## Abstract

Objective: This study was conducted to validate the involvement of the PAR2-PKC-TRPA1 pathway in cough hypersensitivity (CHS) development. Methods: Guinea pigs were divided into a blank control, a citric acid-induced enhanced cough model, and drug intervention groups. The effects of the drugs on capsaicin-induced cough responsiveness in a cough model were observed. The effects of individual and combined treatments (including PAR2 agonists, TRPA1 agonists, PAR2 antagonists, TRPA1 antagonists, PKC agonists, and PKC antagonists) on PAR2, phospho-PKC (pPKC), and TRPA1 expression in bronchial tissues and the vagus ganglion (jugular and nodose) in the cough model and control groups were assessed. Additionally, whole-cell patch-clamp recordings were conducted to evaluate the effects of the drugs on vagus ganglion neuron electrophysiological activity. Results: ① Both PAR2 antagonists and TRPA1 antagonists significantly reduced cough frequency in guinea pigs with a cough, and the PAR2 antagonist inhibited coughing induced by the TRPA1 agonist. ② Western blotting and multiplex immunohistochemistry (mIHC) indicated that PAR2, pPKCα, PKCα, and TRPA1 expression in bronchial and vagus ganglion tissues was elevated in the cough model compared with the control, with TRPA1 expression being regulated by PAR2 and PKC being involved in this regulatory process. ③ Whole-cell patch-clamp recordings demonstrated that TRPA1 agonists induced an inward current in nodose ganglion neurons, which was further amplified by PAR2 agonists; this amplification effect was blocked by PKC antagonist. Additionally, PAR2 antagonists inhibited the inward current induced by TRPA1 agonists. ④ At various concentrations, including the optimal antitussive concentration, PAR2 antagonists did not significantly affect pulse amplitude, arterial oxygen saturation, heart rate, body temperature, or respiratory rate in guinea pigs. Conclusion: PAR2 regulates TRPA1 through PKC in cough syndrome (CHS) pathogenesis, making targeting PAR2 a safe and effective therapeutic strategy for CHS.

## 1. Introduction

Chronic cough is a common respiratory disorder that refers to coughing that lasts more than 8 weeks in the absence of abnormalities on chest radiographs [1,2]. Owing to the diverse causes of chronic cough and the complexity of the clinical manifestations, a significant portion of chronic cough cases are idiopathic or have identifiable causes but suboptimal treatment outcomes; these causes are referred to as refractory cough. Refractory cough is a clinical issue for which treatments are urgently needed. Patients with refractory cough exhibit increased cough sensitivity, a pathological condition recognized as cough hypersensitivity syndrome (CHS) [2].

Cough receptors include myelinated Aδ fiber cough receptors that are sensitive to mechanical stimulation and unmyelinated C afferent nerve fibers that are sensitive to chemical stimulation. They are distributed in the basal layer of epithelial cells in the tracheobronchial mucosa and around sensory nerve endings between cells [2,3]. C fiber endings express various members of the transient receptor potential (TRP) family, which consists of seven subfamilies, including the TRPA, TRPM, and TRPV subfamilies [4,5,6]. Both transient receptor potential ankyrin 1 (TRPA1) and transient receptor potential vanilloid1(TRPV1) are closely associated with cough hypersensitivity (CHS) and are expressed in airway C fibers and the vagus ganglion(jugular and nodose), there is a synergistic effect between TRPA1 and TRPV1 [7,8,9,10]. After TRPA1/TRPV1 is stimulated, the channel opens, resulting in the influx of calcium ions, which generate an action potential and excite C fibers, causing them to produce neuronal inflammatory mediators such as substance P (SP), calcitonin gene-related peptide (CGRP) and simultaneously transmit local excitatory signals to the central nervous system. A cough command is then issued from the central nervous system. Repeated stimulation of TRPA1/TRPV1 leads to peripheral nerve sensitization in the airway, which in turn leads to central nerve sensitization, which is the underlying mechanism of CHS [1,11,12].

Numerous studies on TRPA1 have confirmed its involvement in the development of CHS. Both animal and human experiments have shown that inhalation of the TRPA1 agonist cinnamaldehyde can trigger coughing [5,8]. GDC-0334 [13] and BI01305834 [14], as TRPA1 antagonists are effective in the treatment of asthma- related Airway Hyper Reactivity (AHR) and cough.

Research has confirmed that protease-activated receptor 2 (PAR2), a member of the G protein-coupled receptor family, acts as an upstream regulator of TRPA1. PAR2 is expressed in various cell types, including endothelial cells, epithelial cells, exocrine gland cells, and fibroblasts [15]. It is associated with several conditions, including bronchial asthma, chronic pain, and chronic itch [16,17]. Numerous studies have shown that endogenous and exogenous stimuli can activate PAR2 to cause airway inflammation and airway hyperresponsiveness [18,19,20,21,22]. In guinea pigs, PAR2 and TRPA1 are coexpressed in the vagus ganglion and functionally interact with each other [23]. PAR2 is involved in the occurrence of a variety of neuropathic diseases via the regulation of TRPA1 [23,24,25,26]. PAR2 is also involved in cough formation by regulating TRPV1 [27,28]. Additionally, studies on other neuropathic diseases have shown that PAR2 modulates other cough-related receptors, such as TRPV4 [26] and P2X3 [29]. Therefore, we propose that PAR2, as an upstream regulator of these receptors, may mediate their roles in cough reflex, making it an ideal target for cough treatment. As such, this study focuses on TRPA1, aiming to investigate the role of PAR2 in the development of cough sensitivity via the regulation of TRPA1 in guinea pigs with cough.

## 2. Experiment and Methods

### 2.1. Animals

The animals used in this study were male Dunkin Hartley guinea pigs weighing approximately 300 g (Jiesijie, Shanghai, China), housed in the animal facility of Shanghai Tongji Hospital, under appropriate temperature and humidity conditions. This study was approved by the Ethics Committee of Shanghai Tongji Hospital (Reference Number: Ethics Review 2022-DW-(036)), and strictly adhered to the principles and guidelines outlined in the National Institutes of Health Guide for the Care and Use of Laboratory Animals.

### 2.2. Establishment of a Citric Acid Induced Enhanced Cough Guinea Pig Model and Assessment of the Capsaicin-Induced Cough Response

A citric acid-induced enhanced guinea pig model was established according to methods described previously by our group [30]. After 1-week adaptation, guinea pigs were randomly divided into a cough model group and control group. Conscious guinea pigs were placed in a whole-body plethysmography (WBP) box (EMKA, Bourre, France), and an ultrasonic atomizer (ANP-1000; EMKA, Bourre, France) was used to induce the inhalation of 0.4 M citric acid/saline twice daily in the early afternoon. The number of coughs by each guinea pig over 1 min at the end of each treatment day was recorded using an EMKA pulmonary function test system (iox2.9 analysis system, EMKA, Bourre, France). The modeling period was 15 days.

Referencing our department’s previous method [30], guinea pigs were placed in the whole-body plethysmograph (WBP) to continuously record respiratory pressure changes via pressure sensors and cough sound signals via microphones. Using the dual-chamber respiratory flow analyzer and cough monitoring modules (Emka’s iox3.1 software), we recorded the number of coughs for each guinea pig over 3 min, specifically from 2 min after inhalation of a capsaicin solution (10^–4^ M) to 1 min after cessation of inhalation. A typical cough was defined by the following criteria: head and abdominal movement, quick head extension with mouth opening, rapid abdominal contraction, and the audible cough sound, with the respiratory waveform exceeding twice the baseline amplitude. At the end of monitoring, recorded waveforms were reviewed to exclude interference from sneezing and other physical activities in order to obtain the total cough count.

### 2.3. Drug Intervention

Guinea pigs with cough were intraperitoneally injected with the PAR2 inhibitor or the TRPA1 antagonist 30 min before citric acid inhalation on the 13th to 15th day of model establishment.

In the control group, drugs were administered on the last 3 days (days 13–15). Thirty minutes prior to saline inhalation, the following agents were administered via intraperitoneal injection (combined or alone): the PAR2 agonist, an PAR2 antagonist, the TRPA1 agonist, the PKC agonist and the PKC antagonist. All agonist and antagonist were obtained from MedChemExpress (Nanjing, China). The details are as follows:

PAR2 agonist SLIGRL-NH2 (SLI-NH2): 1 mg was dissolved in DMSO to prepare a stock solution of 1 mg/mL. It was then diluted with saline for guinea pig injection (200 µg/kg).

PAR2 antagonist FSLLRY-NH2 (FSL-NH2): dissolve FSL-NH2 in DMSO to prepare a stock solution of 1 mg/mL, then diluted with saline for injection (200 µg/kg).

TRPA1 agonist cinnamaldehyde (CA): a stock solution of 10 mg/mL was prepared in DMSO and diluted in saline for guinea pig injection (5 mg/kg).

TRPA1 antagonist HC-030031 (HC): a stock solution of 25 mg/mL was prepared in DMSO and diluted in saline for injection (200 mg/kg).

PKC agonist Phorbol 12-myristate 13-acetate (PMA): a stock solution of 1 mg/mL was prepared in DMSO and diluted for injection (2 µg/kg).

PKC antagonist GF109203X (GFX): dissolve GFX in DMSO to prepare a stock solution of 1 mg/mL and diluted for injection (1 µg/kg). The experimental conditions were optimized during the study, and no animals died during the study.

### 2.4. Assessment of Vital Signs

Arterial oxygen saturation (SPO2), heart rate (HR), respiratory rate (RR), pulse amplitude (pulse filling) and respiratory rate (RR) were monitored in guinea pigs with cough and blank controls using a vital sign monitor (MouseOx Plus, Starr Company, Munich, Germany). The body temperature of the guinea pigs was monitored using an infrared thermometer (Gaosong DT-9836, Keyi Technology Co., Ltd., Shenzhen, China). Three measurements for each index were recorded for each guinea pig, and the values were averaged by the software.

### 2.5. Collection of Bronchoalveolar Lavage Fluid (BALF), Bronchial Tissues, and the Vagus Ganglion

A total of 5–10 mL of 1X PBS solution was aspirated using a 10 mL needle, and the fluid was injected into the lung tissue through the trachea via an intubation hose; this process was repeated 3 times. Then, the alveolar lavage fluid was collected in a 15 mL sterilized centrifuge tube (recovery rate > 70%) and placed on ice. Afterward, the lung tissues were separated with tissue scissors and forceps, the tracheobronchial and lung tissues were completely dissected, and the bilateral bronchial carina (including a small amount of lung tissue) was removed and stored in cryotubes in liquid nitrogen. The fur was completely removed from the heads and necks of the guinea pigs, and the vagus ganglion was located, between the ossicles and the underlying mastoid bone. To avoid contamination, the vagus ganglion was separated with microscopic tweezers and microscissors after using new sterile gloves. The connective tissue attached to the surface of the ganglion was quickly removed, and the ganglion was thoroughly cleaned and then stored in cryotubes in liquid nitrogen.

### 2.6. Measurement of SP and CGRP Levels in BALF by ELISA

In accordance with the manufacturer’s instructions, an SP ELISA Kit (Xinqidi, EIA06357Gu, Wuhan, China) and a CGRP ELISA Kit (Xinqidi, EIA05472Gu, Wuhan, China) were used to measure SP and CGRP levels in the BALF.

### 2.7. Western Blotting

Total protein was extracted from jugular and nodose ganglion and trachea tissues. Proteins(30 μg) were separated using 10% sodium dodecyl sulfate–polyacrylamide gel electrophoresis and subsequently transferred to polyvinylidene difluoride (PVDF) membranes (Millipore, Burlington, MA, USA). After blocking with PBS containing 5% skim milk for 2 h at room temperature, the membranes were washed with PBS-T (PBS with 0.05% Tween-20). They were then incubated overnight at 4 °C with rabbit primary antibodies against TRPA1 (1:1000, Novus, LLC, Salt Lake City, UT, USA), PKCα (1:1000, Abcam, Cambridge, UK), phosphorylated PKCα (pPKCα) (1:1000, Cell Signaling Technology, Danvers, MA, USA), or PAR2 (1:1000, Abcam, Cambridge, UK). Following PBS-T washes, the membranes were incubated with horseradish peroxidase (HRP)-conjugated goat anti-rabbit IgG secondary antibody (1:2000, Abcam, Cambridge, UK) for 2 h at room temperature. After additional PBS-T washing, chemiluminescence substrate (Thermo Fisher Scientific, Waltham, MA, USA) was applied to the membranes. Signal detection was performed using an enhanced chemiluminescence system (Image Quant LAS-4000 MINI; GE Healthcare Bioscience, Pittsburgh, PA, USA). Samples from five animals per group were analyzed using Image-Pro Plus software 7.0. Western blot original images can be found in Figure S1.

### 2.8. Isolation and Culture of Nodose Ganglion Cells

Nodose ganglion tissue fragments were digested with 1 mg/mL collagenase type I (Coolaber, Beijing, China) for 35–45 min at 37 °C. Then, 1 mg/mL DNase1 (Coolaber, Beijing, China) was added to digest the DNA for 15–20 min at 37 °C, and the digestion reaction was terminated by adding fetal bovine serum (Gibco, Waltham, MA, USA) stock solution after most neurons were found to be lysed under a microscope. The digested cells were subsequently plated on 0.1 mg/mL poly-L-lysine (Coolaber, Beijing, China)-coated round coverslips, and patch-clamp experiments were performed after most neurons had adhered for 8 h.

### 2.9. Patch-Clamp Recording of Nodose Ganglion Neurons

Individual intact adherent nodose ganglion neurons were selected for recordings, ensuring that at least 10 cells from each group were recorded. A 3D micromanipulator was adjusted to slowly bring a glass electrode into proximity with the cell membrane and achieve membrane rupture to initiate the experimental recordings. After establishing the whole-cell configuration, a liquid-filled recording electrode was brought near the cell. As the electrode approached, its impedance increased, negative pressure was applied, and the holding voltage was set to −70 mV to form a high-resistance seal. Once a stable high-resistance seal was achieved, the negative pressure was released to compensate for electrode capacitance. Mechanical negative pressure was then applied to rupture the cell membrane at the electrode tip, thereby establishing the whole-cell configuration. Experimental drugs were diluted in an extracellular solution and stored in separate syringes, infused into the recording chamber by gravity with rapid solution exchange controlled via switches. All electrophysiological experiments were conducted at room temperature (20–25 °C) using an Axon 700B patch-clamp amplifier for recording, with experimental parameters controlled, data acquisition performed, and the stimulation executed via Clampex 10.7 software.

To investigate the effect of PAR2 activation on TRPA1-induced currents, we first administered 500 µM cinnamaldehyde (TRPA1 agonist) to vagus ganglion neurons from the citric acid induced enhanced cough model group and the blank control group for 20 s. After washout, 100 µM SLIGRL-NH2 (PAR2 agonist) was administered to activate PAR2, followed by TRPA1 agonist administration for 20 s, and the evoked inward currents were recorded. In addition, we observed the effect of PAR2 inhibition on TRPA1-induced currents by adding 10 µM FSLLRY-NH2 (PAR2 antagonist) to neurons to inhibit PAR2 and then adding the TRPA1 agonist for 20 s, and recording evoked inward currents. In this study, 10 µM GF109203X (PKC antagonist) was used to further investigate whether PKC is involved in the PAR2-mediated effect on TRPA1-evoked currents.

### 2.10. Multiplex Immunohistochemistry and Laser-Scanning Confocal Microscopy

A freezing microtome (CM1520; Leica Biosystems, Shanghai, China) was used, and 4 mm-thick tracheal and vagus ganglion tissue sections were prepared and stored at room temperature (25 °C) for 30 min. Then, the sections were treated with 100 μL of 3% H_2_O_2_, incubated for 10 min (25 °C), and then immersed in PBS-T 3 times for 3 min each. The PBS solution was removed, 100 μL of 5% goat serum (blocking solution) was added to each section, the samples were incubated for 30 min (25 °C), and then the blocking solution was removed. The sections were incubated with a rabbit anti-TRPA1 primary antibody(1:1000, Novus, LLC, Centennial, CO, USA), a rabbit anti-pPKCα primary antibody (1:1000, Cell Signaling Technology, Danvers, MA, USA), a rabbit anti-PAR2 primary antibody PAR2 (1:1000, Abcam, Cambridge, UK) and a mouse anti-PGP9.5 primary antibody (1:200, Abcam, Cambridge, UK) for 30 min, followed by incubation with FITC-conjugated rabbit anti-goat IgG (1:500; Proteintech, Rosemont, IL, USA) at 37 °C for 30 min. Nuclei were stained with DAPI (1:1000; Thermo Fisher Scientific, Waltham, MA, USA), use a laser scanning confocal microscope (Nikon Eclipse TI; Nikon, Tokyo, Japan) to observe and analyze the sections. To ensure objectivity, an investigator who was blinded to the experimental procedures randomly selected six different microscopic fields of bronchial and epithelial cross sections from each sample. Each field size was similar, the scale was 50 mm. The intensities of the red (PAR2), green (pPKCα), pink (TRPA1), and orange (PGP9.5) fluorescence signals in each image were measured by Image-Pro Plus 6.0 software (Media Cybernetics, Rockville, MD, USA). Data were collected simultaneously for each group under the same conditions.

### 2.11. Statistical Analysis

Data are presented as means ± SEM. Group differences were assessed using two-tailed, unpaired Student’s t-tests. Pearson correlation analysis was employed to evaluate correlations between continuous variables. Statistical analyses were conducted using GraphPad Prism 7.0 software (GraphPad Software, La Jolla, CA, USA), with a *p*-value of <0.05 denoting statistical significance. A power analysis approach was used to calculate the sample size.

## 3. Results

### 3.1. Cough Sensitivity to Different Drugs

**Figure 1:**
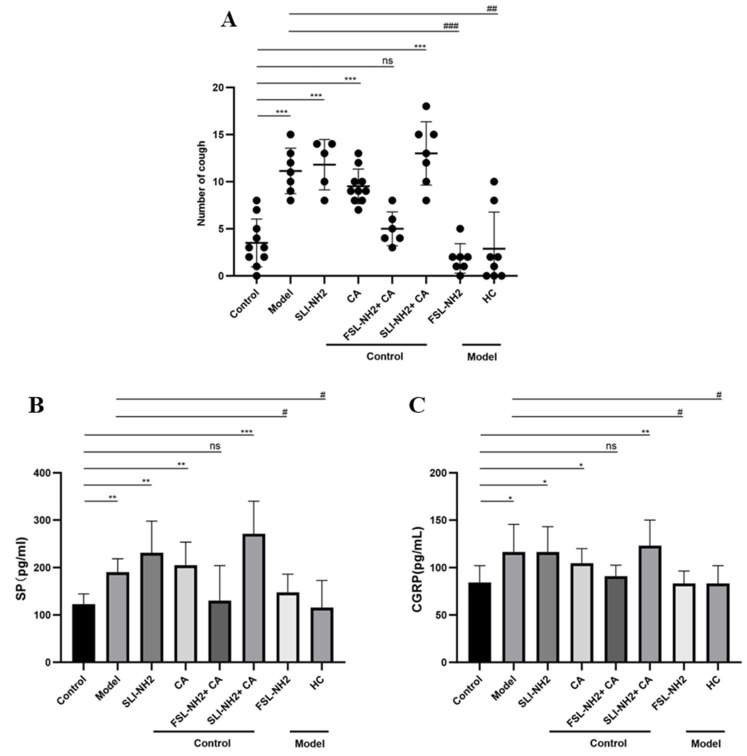
Effects of the PAR2,TRPA1 agonist/antagonist on cough sensitivity in guinea pigs with cough induced by capsaicin. (**A**) In citric acid-induced enhanced cough guinea pigs, there was a significant increase in cough frequency. However, the cough frequency in the guinea pigs with a cough was not further increased by the activation of PAR2/TRPA1. Inhibition of PAR2 or TRPA1 significantly reduced the cough frequency in guinea pigs with a cough. (**B**) SP levels in the BALF of guinea pigs with a cough were further increased by the activation of PAR2. Inhibition of PAR2/TRPA1 decreased the level of SP in the BALF of guinea pigs with a cough. (**C**) The changes in CGRP levels in the BALF of guinea pigs were consistent with the changes in SP levels shown in (**B**). PAR2 agonist SLIGRL-NH2 (SLI-NH2 200 µg/kg), TRPA1 agonist cinnamaldehyde (CA 5 mg/kg), PAR2 antagonist FSLLRY-NH2 (FSL-NH2 200 µg/kg), TRPA1 antagonist HC-030031 (HC 200 µg/kg). The results are expressed as means ± standard deviations (vs. the control group: * *p* < 0.05, ** *p* < 0.01, *** *p* < 0.001; vs. the cough model group: # *p* < 0.05, ## *p* < 0.01, ### *p* < 0.001; ns: p > 0.05).

In the model group, the number of coughs by each guinea pig significantly increased (11.14 ± 2.41 vs. 3.50 ± 2.55, *p* < 0.001). The activation of PAR2 in guinea pigs led to a notable increase in cough frequency (11.80 ± 2.68 vs. 3.50 ± 2.55, *p* < 0.001), as did the activation of TRPA1 (9.50 ± 1.84 vs. 3.50 ± 2.55, *p* < 0.001). Inhibition of PAR2 resulted in a significant reduction in cough frequency in guinea pigs with a cough (1.86 ± 1.57 vs. 11.14 ± 2.41, *p* < 0.001), as did inhibition of TRPA1 (2.88 ± 1.38 vs. 11.14 ± 2.41, *p* < 0.001) (Figure 1A).

### 3.2. SP and CGRP Levels in BALF

The levels of the neurogenic inflammatory factors SP (*p* = 0.002) and CGRP (*p* = 0.045) were elevated in guinea pigs with a cough compared with control guinea pigs. Activation of PAR2 resulted in increased levels of SP (*p* = 0.002) and CGRP (*p* = 0.035) in guinea pigs, and activation of TRPA1 also led to elevated SP (*p* = 0.002) and CGRP (*p* = 0.049) levels. Conversely, inhibition of PAR2 significantly reduced SP (*p* < 0.001) and CGRP (*p* = 0.048) levels in the BALF of guinea pigs with a cough. Similarly, inhibition of TRPA1 resulted in decreased SP (*p* < 0.001) and CGRP (*p* = 0.042) levels (Figure 1B,C).

### 3.3. Assessment of Vital Signs After PAR2 Antagonist Application

Different concentrations of the PAR2 antagonist FSLLRYNH2, including the optimal antitussive concentration (200 µg/kg), had no significant effects on pulse amplitude, arterial oxygen saturation, heart rate, body temperature, or respiratory rate in awake guinea pigs (Table 1).

### 3.4. PAR2/PKC/TRPA1 Expression on Peripheral Nerve Fibers and Neurons in Guinea Pigs with a Cough

**Figure 2:**
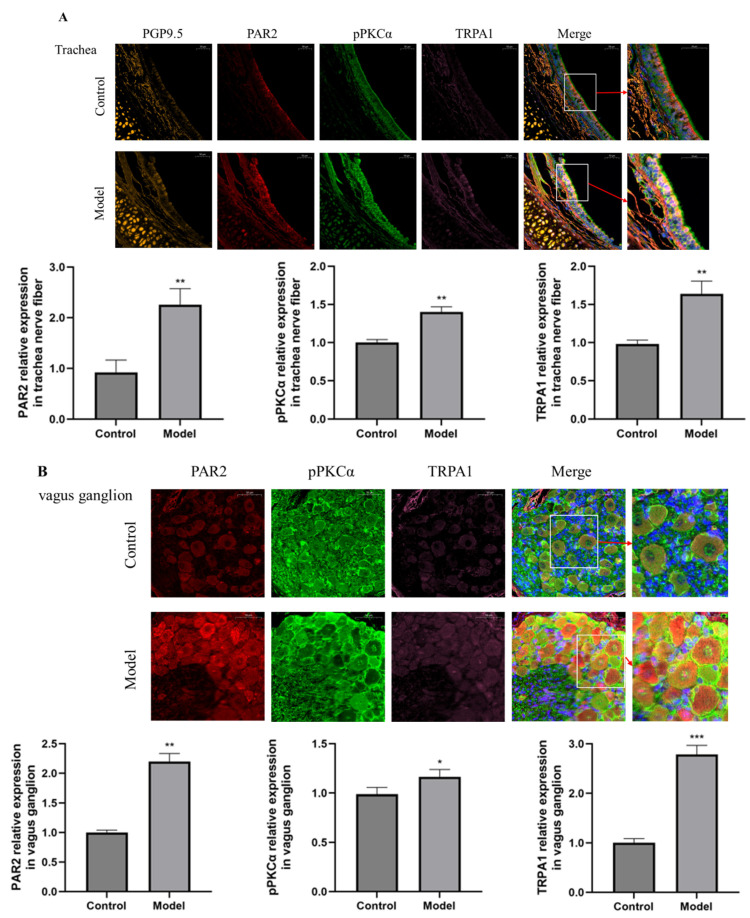
Immunofluorescence was used to detect the expression of PAR2 (red fluorescence), pPKCα (green fluorescence), TRPA1 (pink fluorescence) and PGP9.5 in nerve fibers (orange fluorescence) in the trachea and vagus ganglion (scale = 50 μm). (**A**) The expression of PAR2, pPKCα and TRPA1 in the trachea was greater in the citric acid-induced enhanced cough group than in control guinea pigs. (**B**) The expression levels of PAR2, pPKCα and TRPA1 in the vagus ganglion were greater in cough group than in control guinea pigs. The results are expressed as the means ± standard deviations (vs. the control group: * *p* < 0.05, ** *p* < 0.01, *** *p* < 0.001).

Immunofluorescence staining revealed that the expression of PAR2, pPKC and TRPA1 in nerve fibers in the trachea and vagus ganglion neurons was significantly increased in guinea pigs with a cough compared with control guinea pigs (Figure 2).

### 3.5. PAR2 Regulates TRPA1

**Figure 3:**
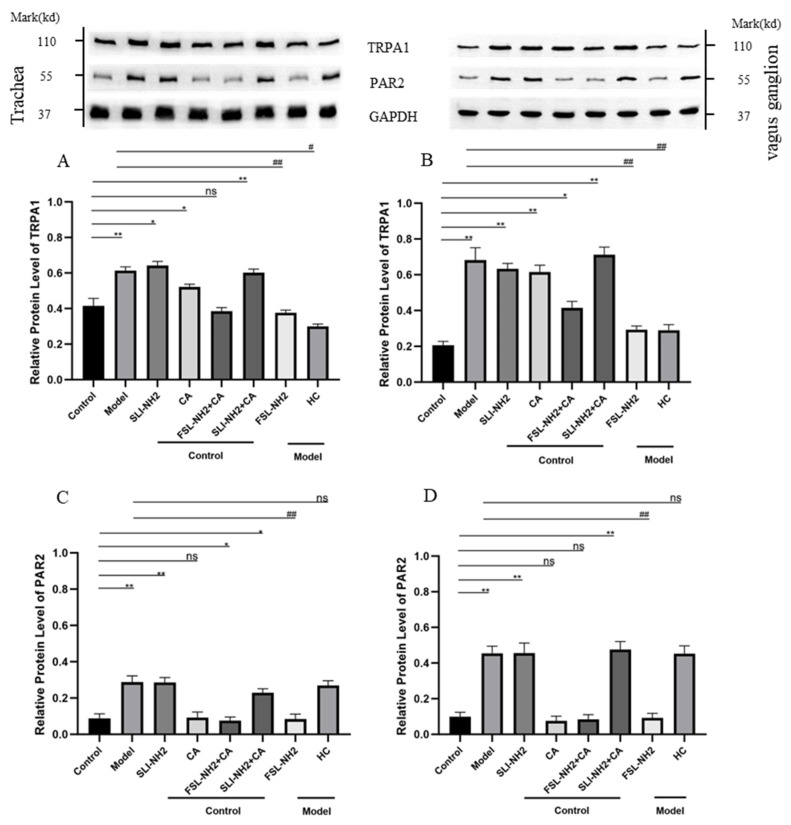
The effects of the PAR2, TRPA1agonist/antagonist on the expression of PAR2 and TRPA1 in nerve fibers in the trachea and the vagus ganglion of guinea pigs were evaluated. (**A**) The expression of TRPA1 in the nerve fibers of the trachea was increased in the citric acid-induced enhanced cough group after drug treatment. Activation of PAR2 upregulated TRPA1 expression, whereas inhibition of PAR2 in cough group suppressed TRPA1 expression. (**B**) The expression of TRPA1 in the vagus ganglion after drug treatment tended to be similar to that in (**A**). (**C**) The expression of PAR2 in the nerve fibers of the trachea was increased in the cough group after drug treatment, whereas TRPA1 receptor activation/inhibition had no effect on PAR2 expression. (**D**) The expression of PAR2 in the vagus ganglion after drug treatment tended to be consistent with that in (**C**). PAR2 agonist SLIGRL-NH2 (SLI-NH2 200 µg/kg), TRPA1 agonist cinnamaldehyde (CA 5 mg/kg), PAR2 antagonist FSLLRY-NH2 (FSL-NH2 200 µg/kg), TRPA1 antagonist HC-030031 (HC 200 µg/kg). The results are presented as means ± standard deviations (vs. the control group: * *p* < 0.05, ** *p* < 0.01; vs. the cough model group: # *p* < 0.05, ## *p* < 0.01; ns: *p* > 0.05).

In nerve fibers in the trachea, TRPA1 expression was regulated by PAR2. The increase in TRPA1 expression in the citric acid induced enhanced cough group and the TRPA1 agonist group could be inhibited by a PAR2 antagonist. In vagus ganglion, the changes in the expression of PAR2 and TRPA1 were essentially consistent with those in nerve fibers in the trachea (Figure 3).

**Figure 4:**
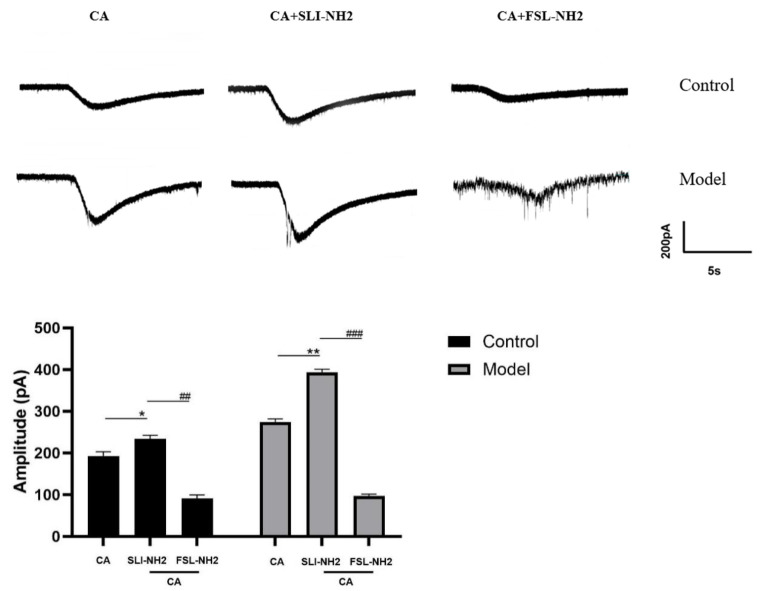
Effects of a PAR2 agonist, a TRPA1 agonist and a PAR2 antagonist on the activity of guinea pig nodose ganglion neurons. Activation of TRPA1 induced an inward current, activation of PAR2 further amplified the inward current elicited by the TRPA1 agonist, and inhibition of PAR2 reduced the inward current elicited by the TRPA1 agonist. PAR2 agonist (SLI-NH2 100 µM), TRPA1 agonist (CA 500 µM), PAR2 antagonist (FSL-NH2 10 µM). The results are presented as the means ± standard deviations (vs. the TRPA1 agonist group: * *p* < 0.05, ** *p* < 0.0; vs. the PAR2 agonist + TRPA1 agonist group: ## *p* < 0.01, ### *p* < 0.001).

To further investigate the relationship between PAR2 and TRPA1 and its effect on electrophysiology activity, we performed the patch–clamp recordings of nodose ganglion neurons in control and guinea pigs with a cough (Figure 4). After the administration of the TRPA1 agonist, an inward current was observed. The amplitude of the current change in the TRPA1 agonist group was greater than that in the blank control group. Compared with the TRPA1 agonist alone, the combination of the PAR2 agonist and TRPA1 agonist further increased the inward current (192.67 ± 10.50 vs. 234.11 ± 8.89 pA, *p* < 0.05). This effect was more obvious in guinea pigs with a cough (274.33 ± 7.51 vs. 394.01 ± 7.21 pA, *p* < 0.01). The PAR2 antagonist reduced the amplitude of the inward current evoked by the TRPA1 agonist (234.11 ± 8.89 vs. 91.33 ± 8.02 pA, *p* < 0.01), and this effect was also observed in guinea pigs with a cough (394.01 ± 7.21 vs. 97.01 ± 4.58 pA, *p* < 0.001). It can be concluded that the PAR2 agonist amplified cellular activity evoked by TRPA1. Moreover, the PAR2 antagonist inhibited TRPA1 agonist-induced cellular activity to some extent.

### 3.6. PAR2 Regulates Cough Hypersensitivity Through TRPA1 via a Mechanism Involving PKC

In the previous sections of this study, we detected that the expression of PAR2, PKC and TRPA1 in the trachea and vagus ganglion of guinea pigs with cough was increased; according to this finding, combined with those of previous studies [27], we speculated that PAR2 further regulates TRPA1 through PKC to participate in CHS.

**Figure 5:**
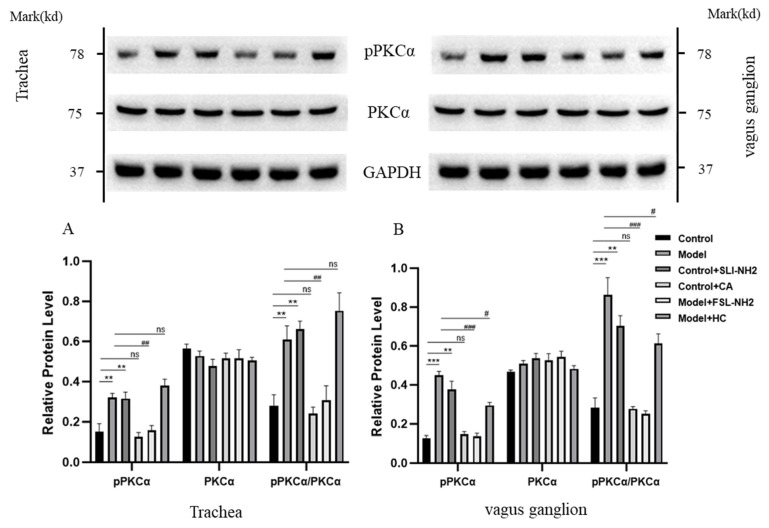
The effects of a PAR2,TRPA1 agonist/antagonist on the phosphorylation of PKCα (pPKCα) and PKCα in the nerve fibers of the trachea and the vagus ganglion in guinea pigs were evaluated. (**A**) In the nerve fibers of the trachea in the citric acid-induced enhanced cough guinea pigs, pPKCα was increased. Activation or inhibition of PAR2 upregulated or downregulated pPKCα, respectively, whereas activation or inhibition of TRPA1 did not affect pPKCα. (**B**) The change in pPKCα expression in the vagus ganglion was consistent with that in (**A**). PAR2 agonist (SLI-NH2, 200 µg/kg), TRPA1 agonist (CA, 5 mg/kg), PAR2 antagonist (FSL-NH2, 200 µg/kg), TRPA1 antagonist (HC, 200 µg/kg). The results are presented as the means ± standard deviations (vs. the control group: ** *p* < 0.01, *** *p* < 0.001; vs. the cough model group: # *p* < 0.05, ## *p* < 0.01, ### *p* < 0.001; ns: p > 0.05).

In the nerve fibers in the trachea, the phosphorylation of PKCα (pPKCα) was significantly greater in guinea pigs with a cough than in control guinea pigs (*p* < 0.001) (Figure 5A). The pPKCα after PAR2 activation was greater than that in the control group (*p* < 0.05). The PAR2 antagonist significantly reduced the pPKCα in cough guinea pigs (*p* < 0.001). However, the pPKCα was not significantly affected by the TRPA1 agonist. The changes in pPKCα in the vagus ganglion were essentially consistent with those in nerve fibers in the trachea (Figure 5B).

**Figure 6:**
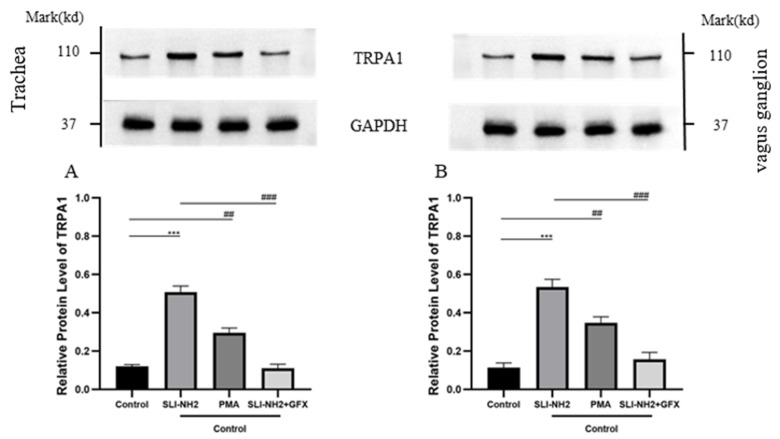
The effects of a PAR2, PKC agonist/antagonist on the expression of TRPA1 in the nerve fibers of the trachea and vagus ganglion in guinea pigs were evaluated. (**A**) In the nerve fibers of the trachea, activation of PAR2 or PKC led to upregulation of TRPA1 expression. Inhibition of PKC blocked the upregulation of TRPA1 induced by the PAR2 agonist. (**B**) The change in TRPA1 expression in the vagus ganglion was consistent with that observed in (**A**). PAR2 agonist (SLI-NH2, 200 µg/kg), PKC agonist (PMA, 2 µg/kg), PKC antagonist (GFX, 1 µg/kg). The results are presented as the means ± standard deviations (vs. the control group: *** *p* < 0.001; vs. the cough model group: ## *p* < 0.01, ### *p* < 0.001).

To further verify the relationships among PKC, PAR2 and TRPA1, we examined the effects of PKCα on TRPA1 protein expression in nerve fibers in the trachea and vagus ganglion of guinea pigs. The results revealed that TRPA1 expression was upregulated after PKC activation and that the upregulation of TRPA1 induced by PAR2 activation could be inhibited by the PKC antagonist (Figure 6).

**Figure 7:**
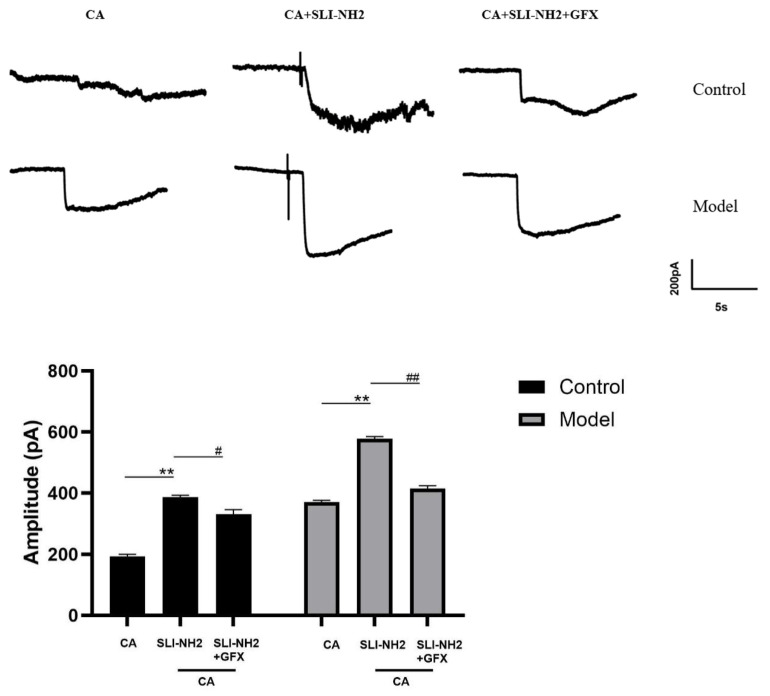
The effects of a PAR2 agonist, TRPA1 agonist, and PKC antagonist on the electrical activity of neurons in the nodose ganglion of guinea pigs were evaluated. Activation of TRPA1 resulted in an inward current, the amplitude of which was further increased by the PAR2 agonist in the presence of the TRPA1 agonist. Additionally, the amplitude of the inward current in the citric acid-induced enhanced cough group was significantly greater than that in the control group. A PKC antagonist effectively blocked this effect. PAR2 agonist (SLI-NH2 100 µM), TRPA1 agonist (CA 500 µM), PKC antagonist (GFX 10 µM). The results are presented as the means ± standard deviations (vs. the CA group: ** *p* < 0.01; vs. the SLI-NH2 + CA group: # *p* < 0.05, ## *p* < 0.01).

In the previous sections of this study, we confirmed that PKC is involved in the development of cough hypersensitivity and that its expression is regulated by PAR2, which in turn modulates TRPA1 expression. To further validate the influence of PAR2 on TRPA1 through PKC, we performed patch-clamp recordings of vagus ganglion neurons in both control guinea pigs and guinea pigs with a cough (Figure 7). After administration of a TRPA1 agonist, inward currents were observed, and the amplitude was greater in guinea pigs with a cough than in control guinea pigs. The application of a PAR2 agonist in the presence of the TRPA1 agonist further increased the amplitude of the inward current (193.67 ± 6.66 vs. 386.33 ± 6.66 pA, *p* < 0.01), with a more pronounced increase in guinea pigs with a cough (370.33 ± 6.11 vs. 577.31 ± 7.63 pA, *p* < 0.01). The amplitude of the inward current significantly decreased in the group treated with a PKC antagonist in combination with the TRPA1 and PAR2 agonists compared with the group treated with only the TRPA1 and PAR2 agonists (386.33 ± 6.66 vs. 331.01 ± 14.53 pA, *p* < 0.05), and this effect was also observed in guinea pigs with a cough (577.31 ± 7.63 vs. 414.34 ± 9.07 pA, *p* < 0.001). These results indicate that the inward current induced by the TRPA1 agonist in response to the PAR2 agonist can be partially blocked by PKC.

## 4. Discussion

This study demonstrated that PAR2 can upregulate TRPA1 expression in the airway and vagal ganglia via pPKC and that this effect is accompanied by increased levels of SP and CGRP in BALF. Additionally, changes in vagal neuron currents confirmed that the PAR2–PKC–TRPA1 pathway increasing vagal excitability, contributing to the development of chronic CHS.

TRPA1, a transient receptor potential ion channel, induces calcium influx upon activation. Experiments have shown that TRPA1 channels can be activated by cold stimuli (temperatures below 17 °C) [31,32] and can also be activated by chemical stimuli, such as inflammatory stimuli [33]. Previous animal and human studies have confirmed the crucial role of TRPA1 in cough [5,9,34]. Inhalation of the TRPA1 agonist cinnamaldehyde activates TRPA1 receptors on airway sensory C fibers, leading to coughing. These C fibers are sensitive to various endogenous and exogenous mediators. Upon stimulation, nerve endings become excited, resulting in the release of multiple neurogenic inflammatory mediators, including SP and CGRP [34,35,36]. In this study, this phenomenon was confirmed. The role of TRPA1 in cough has been confirmed [13,14].

TRPA1 and TRPV1 co-express in the cough pathway and exhibit a positive interaction [8,10]. Studies have confirmed that PAR2 can regulate TRPV1 to participate in post-infection cough [28], and PAR2 can also regulate the cough representation of guinea pigs by affecting TRPV1 [27]. In neuropathic pain research, in animal experiments, inhibition of PAR2 and TRPA1 signals increases neuropathic pain evoked by chemotherapeutic bortezomib [25,26]. In rat models of spinal cord injury, PAR2 antagonists can inhibit pain by inhibiting TRPA1 [24]. PAR2 is involved in the amplification of the motor pressor reflex in rats with femoral artery ligation by regulating TRPA1 [23]. These results suggest that PAR2 may also participate in the formation of CHS by regulating TRPA1 in cough.

Therefore, we focused on PAR2, which is upstream of TRPA1. The aim of this study is to verify that PAR2 is also involved in the formation of CHS by regulating TRPA1 from cough manifestations, histological and cytological levels. PAR2, a protease-activated receptor (PAR) subtype and a G protein-coupled receptor, mediates the occurrence and development of various sensory neuropathies [15,21,22,24,26,27]. In the vagus ganglion of guinea pigs, approximately 91% of TRPA1-positive cells are neurons, and TRPA1 is colocalized with PAR2 in 80% of these neurons. These data strongly suggest a functional interaction between PAR2 and TRPA1 in the vagus ganglion [37]. This study also confirmed that PAR2 regulates TRPA1 in second-order neurons in cough hypersensitivity (i.e., neurons in the vagus [38] ganglion). We found that increased cough sensitivity in guinea pigs was accompanied by the upregulation of PAR2 expression in both the airway and vagus nerve ganglia. Furthermore, PAR2 modulates TRPA1, indicating that it plays roles in upstream and downstream mechanisms of cough. Our research demonstrated that the activity of the PAR2-TRPA1 pathway is increased not only in airway tissues but also in vagus nerve ganglia, indicating its involvement in the entire cough process from peripheral to central sensitization.

The mechanisms by which PAR2 sensitizes TRPA1 are diverse; according to most previous studies [39,40], PAR2 sensitizes TRPA1 in a PKC-dependent manner. As a class of enzymes that play a key role in cell signal transduction, PKC can regulate various neural functions, including neurotransmitter release, signal transduction, and receptor sensitization, thereby sensitizing and exciting neurons [41,42]. Additionally, PKC is associated with various chronic airway diseases [38,43,44], and the translocation of PKC to the plasma membrane is involved in the Ca^2+^-dependent regulation of airway smooth muscle, which is closely associated with airway hyperreactivity in asthma [44]. The activation of PKC depends on its phosphorylation [44]. PKC includes many isoforms [45], among the many isoforms of PKC, PKCα may be more closely related to bronchopulmonary C-fibers leading to excessive sensations and reflexes [46], so we chose this subtype for our study. This study confirmed the involvement of PKCα in the development of cough hypersensitivity in guinea pigs through in vivo experiments. In guinea pigs with cough, the level of PKC activation (pPKCα) was increased in the trachea and vagal ganglia. Activation of the PAR2-mediated cough hypersensitivity response was associated with the activation of PKC in the airway and vagal ganglia. PKC activation led to increased TRPA1 expression in these tissues, whereas PKC inhibition blocked PAR2-induced TRPA1 upregulation. Therefore, we propose that PAR2 activates PKC, which further regulates TRPA1, contributing to cough hypersensitivity in guinea pigs. We utilized the “gold standard” patch–clamp technique to validate the function of this pathway at the cellular level. Owing to the unique structure of PAR2, its activation does not directly generate action potentials in the sensory ganglia [47]. Therefore, by preactivating TRPA1, we observed that TRPA1 could induce inward currents in guinea pig vagal ganglion neurons. The activation of TRPA1 increases calcium permeability, leading to calcium influx that triggers action potentials, which is the ionic mechanism underlying enhanced cough signaling. Furthermore, PAR2 agonists can amplify this inward current, confirming the regulatory effect of PAR2 on TRPA1. On this basis, the application of the PKC antagonist suppressed the inward current, indirectly confirming that sensitization of TRPA1 by PAR2 can be blocked by PKC. Additionally, other studies have shown that PAR2 can sensitize TRPA1 through pathways involving phospholipase C (PLC), protein kinase A (PKA), and others, thereby contributing to neuropathic diseases [26].

This study confirms the critical role of PAR2 in the development of CHS. Additionally, we assessed the effects of varying concentrations of the PAR2 antagonist (FSLLRY-NH2) on the vital signs of awake guinea pigs, including pulse amplitude (indirectly reflecting blood pressure), blood oxygen saturation, heart rate, respiratory rate, and body temperature. Compared with those of guinea pigs in the control group, the vital signs of guinea pigs treated with various concentrations of PAR2 antagonists, including the optimal antitussive concentration, were not significantly affected. Thus, we preliminarily validated the safety of the PAR2 antagonist.

## 5. Conclusions

In addition to the involvement of PAR2 in the development of CHS via the PKC-mediated regulation of TRPA1, as mentioned in the present study, PAR2 further amplifies the increase in cough frequency induced by TRPV1 in guinea pigs [27]. Moreover, PAR2 can also participate in the occurrence of other neuropathic diseases by regulating TRPV1, TRPV4, and the ATP receptor P2X3 [26,28,29], and these receptors have been confirmed to be closely related to cough hypersensitivity. Therefore, whether the inhibition of PAR2 can inhibit these receptors and exert a more ideal antitussive effect is also worthy of study. In conclusion, inhibition of PAR2 is an effective and safe strategy for the treatment of CHS, and the development of drugs targeting PAR2 for the treatment of refractory chronic cough is a promising direction for clinical research.

## Figures and Tables

**Table 1:** Effects of gradient concentrations of PAR2 antagonists on vital signs in guinea pigs.

Parameter	Control	FSL-NH2 (µg/kg)				*p* Value
		50	100	200	400	
Breathing Rate (brpm)	85.50 ± 13.65	85.70 ± 15.19	85.02 ± 13.02	87.10 ± 14.67	85.20 ± 16.14	0.998
Pulse Distention (µm)	112.10 ± 23.77	105.40 ± 19.72	97.71 ± 18.96	107.50 ± 18.96	104.50 ± 21.71	0.652
Body Temperature (°C)	38.53 ± 0.89	38.06 ± 0.85	38.63 ± 0.76	38.35 ± 0.74	38.32 ± 0.81	0.578
Heart Rate (bpm)	210.00 ± 19.89	220.60 ± 30.80	208.20 ± 22.71	223.80 ± 30.44	218.90 ± 28.27	0.630
Oxygen Saturation (%)	94.79 ± 2.99	95.00 ± 2.89	94.68 ± 2.98	94.49 ± 2.25	94.59 ± 3.02	0.995

Note: The PAR2 antagonist FSL-NH2 was administered at the following concentrations: 50 µg/kg, 100 µg/kg, 200 µg/kg, and 400 µg/kg. The results are expressed as means ± standard deviations.

## Data Availability

The datasets produced in this study can be obtained by contacting the corresponding author.

## Supplementary Materials

The following supporting information can be downloaded at: https://www.mdpi.com/article/10.3390/biom15020208/s1, Figure S1: The original images for Western blots.
