# Supplementary figures and images for: PAR2 Participates in the Development of Cough Hypersensitivity in Guinea Pigs by Regulating TRPA1 Through PKC
Source: Biomolecules. 2025 Feb 1;15(2):208. doi: 10.3390/biom15020208 (PMC11853178; doi:10.3390/biom15020208)

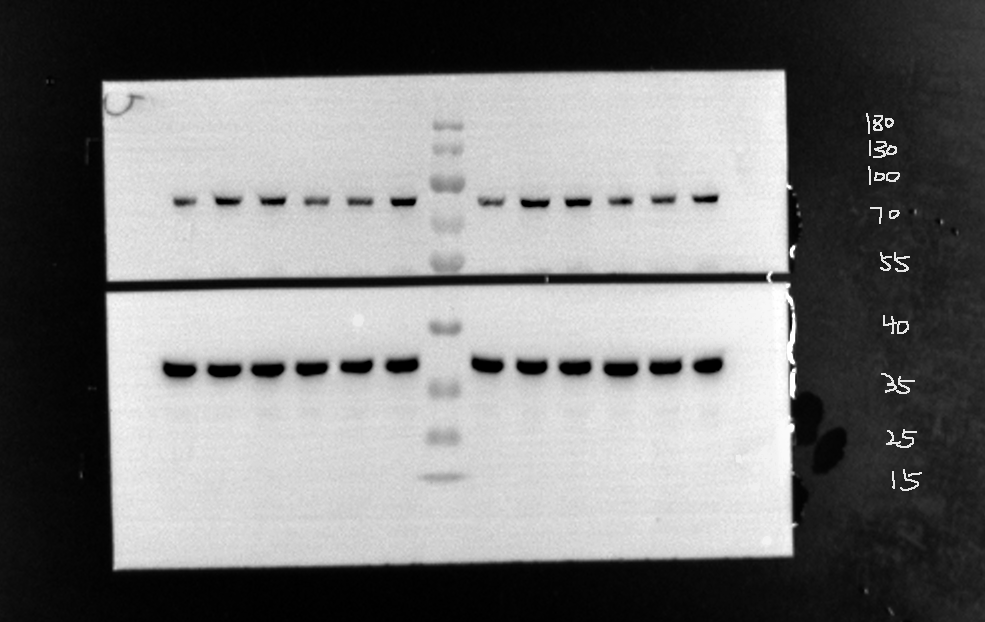

Supplement: Supplementary file 1 [file biomolecules-15-00208-s001.zip › Fig5-2.Tif]

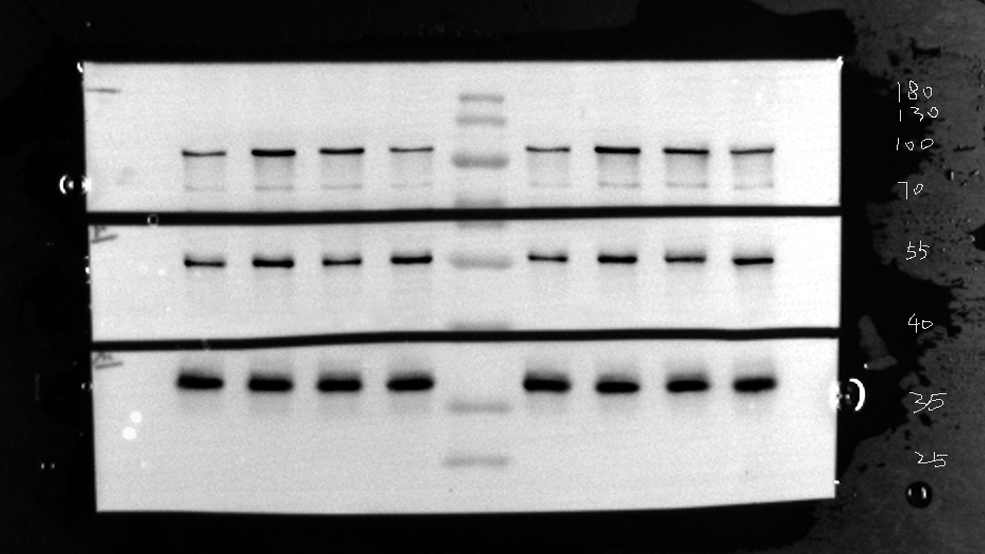

Supplement: Supplementary file 1 [file biomolecules-15-00208-s001.zip › Fig 6.Tif]

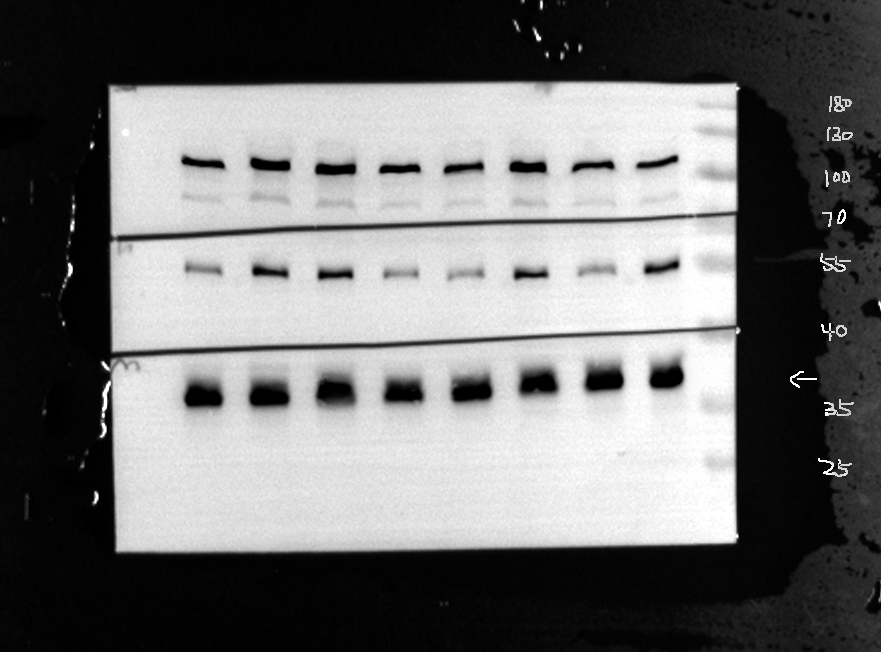

Supplement: Supplementary file 1 [file biomolecules-15-00208-s001.zip › Fig3 A.Tif]

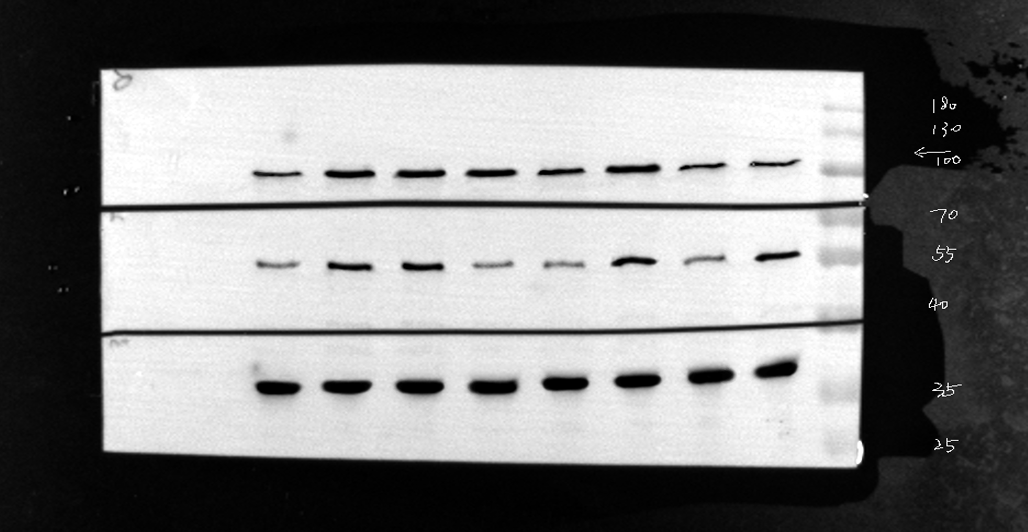

Supplement: Supplementary file 1 [file biomolecules-15-00208-s001.zip › Fig3 B.Tif]

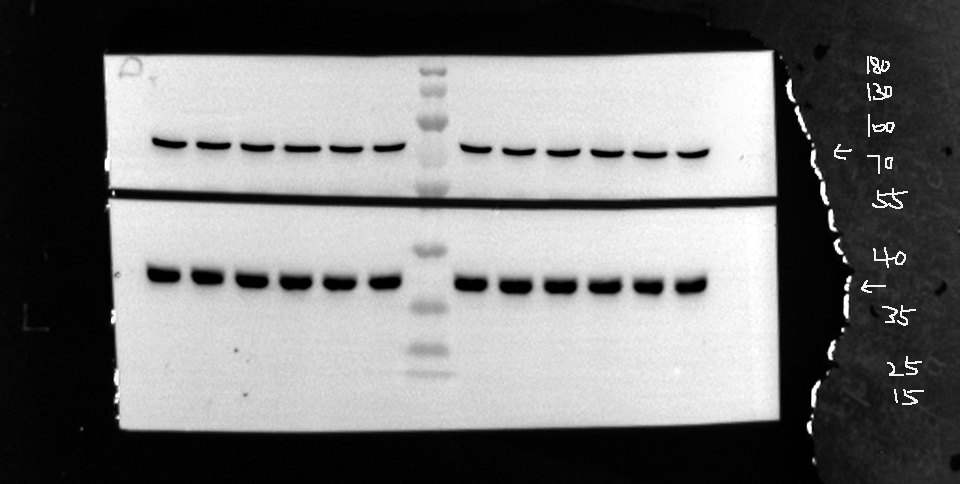

Supplement: Supplementary file 1 [file biomolecules-15-00208-s001.zip › Fig5-1.Tif]
